# *trans*-*N*,*N*,*N*′,*N*′-Tetra­kis(carboxy­meth­yl)cyclo­hexane-1,2-diammonium tetra­chloridocadmium(II) tetra­hydrate

**DOI:** 10.1107/S160053680804110X

**Published:** 2008-12-10

**Authors:** Ping Lian, Qiao-Sheng Hu, Yong-Rong Xie, Hong-Xu Guo

**Affiliations:** a College of Chemistry and Life Science, Gannan Normal University, Ganzhou, Jiangxi 341000, People’s Republic of China; b Department of Chemistry and Environmental Science, Zhangzhou Normal University, Zhangzhou, Fujian 363000, People’s Republic of China

## Abstract

In the title compound, (C_14_H_24_N_2_O_8_)[CdCl_4_]·4H_2_O, the Cd atom in the tetra­hedral [CdCl_4_]^2−^ anion lies on a twofold rotation axis, and the diprotonated organic mol­ecule, *trans*-*N*,*N*,*N*′,*N*′-tetra­kis(carb­oxy­meth­yl)cyclo­hexane-1,2-diammon­ium, has 2 symmetry with the twofold rotation axis running through the mid-point of two C—C bonds in the cyclo­hexane unit. In the crystal structure, classical intra­molecular O—H⋯O and N—H⋯O and inter­molecular O—H⋯O, N—H⋯O, O—H⋯Cl and C—H⋯Cl hydrogen bonds are observed.

## Related literature

For the structure of 1,2-diamino­cyclo­hexane-*N,N′-*tetra­acetate ferrate(III), see: Seibig & Van Eldik (1998 ▶). For related tetra­acetate-based Cu(II) dimeric and polymeric complexes, see: Wang *et al.* (1999 ▶); Ben Amor & Jouini (1999 ▶). For highly stable chiral three-dimensional cadmium 1,2,4-benzene­tricarboxyl­ate structures with NLO and fluorescence properties, see: Wang *et al.* (2006 ▶). For a flexible multicarboxyl­ate ligand used to form homochiral helical Zn and Cd coordination polymers, see: Zang *et al.* (2006 ▶).
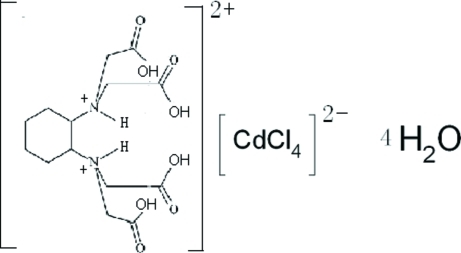


## Experimental

### 

#### Crystal data

(C_14_H_24_N_2_O_8_)[CdCl_4_]·4H_2_O
*M* *_r_* = 674.63
Monoclinic, 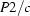

*a* = 11.3772 (14) Å
*b* = 8.5734 (10) Å
*c* = 16.2189 (16) Å
β = 124.119 (6)°
*V* = 1309.7 (3) Å^3^
*Z* = 2
Mo *K*α radiation
μ = 1.30 mm^−1^
*T* = 291 (2) K
0.68 × 0.54 × 0.28 mm

#### Data collection

Siemens SMART CCD area-detector diffractometer
Absorption correction: multi-scan (*SADABS*; Sheldrick, 1996 ▶) *T*_min_ = 0.472, *T*_max_ = 0.712
12160 measured reflections
2400 independent reflections
2319 reflections with *I* > 2σ(*I*)
*R*_int_ = 0.021

#### Refinement

*R*[*F* ^2^ > 2σ(*F* ^2^)] = 0.025
*wR*(*F* ^2^) = 0.069
*S* = 1.01
2400 reflections
166 parameters
9 restraints
H atoms treated by a mixture of independent and constrained refinement
Δρ_max_ = 0.33 e Å^−3^
Δρ_min_ = −0.59 e Å^−3^

### 

Data collection: *SMART* (Siemens, 1996 ▶); cell refinement: *SAINT* (Siemens, 1996 ▶); data reduction: *SAINT*; program(s) used to solve structure: *SHELXS97* (Sheldrick, 2008 ▶); program(s) used to refine structure: *SHELXL97* (Sheldrick, 2008 ▶); molecular graphics: *SHELXTL/PC* (Sheldrick, 2008 ▶); software used to prepare material for publication: *SHELXL97* and *PLATON* (Spek, 2003 ▶).

## Supplementary Material

Crystal structure: contains datablocks I, global. DOI: 10.1107/S160053680804110X/si2140sup1.cif

Structure factors: contains datablocks I. DOI: 10.1107/S160053680804110X/si2140Isup2.hkl

Additional supplementary materials:  crystallographic information; 3D view; checkCIF report

## Figures and Tables

**Table 1:** Hydrogen-bond geometry (Å, °)

*D*—H⋯*A*	*D*—H	H⋯*A*	*D*⋯*A*	*D*—H⋯*A*
O1*W*—H1*WA*⋯O3^i^	0.84 (4)	2.34 (4)	2.970 (3)	132 (5)
N1—H1*B*⋯O2	0.91	2.27	2.750 (3)	112
N1—H1*B*⋯O2^ii^	0.91	2.04	2.857 (2)	149
O1—H1*C*⋯O1*W*	0.92	1.70	2.590 (4)	162
O2*W*—H2*WA*⋯O1*W*^iii^	0.84 (3)	2.24 (3)	2.993 (4)	151 (5)
O2*W*—H2*WB*⋯Cl1^iv^	0.82 (3)	2.51 (4)	3.144 (3)	136 (4)
O1*W*—H1*WB*⋯Cl1^v^	0.86 (7)	2.45 (3)	3.227 (3)	152 (6)
O4—H4*C*⋯O2*W*^vi^	0.83	1.75	2.535 (3)	157
C1—H1*A*⋯Cl2^vii^	0.98	2.67	3.637 (3)	171
C4—H4*A*⋯Cl2^ii^	0.97	2.64	3.600 (2)	170
C4—H4*B*⋯Cl2^v^	0.97	2.83	3.610 (3)	138
C6—H6*A*⋯Cl1^ii^	0.97	2.60	3.537 (2)	163

Symmetry codes: (i) 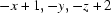; (ii) 
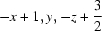
; (iii) 
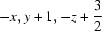
; (iv) 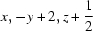; (v) 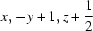; (vi) 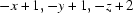; (vii) 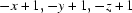.

**Fig. 1.**
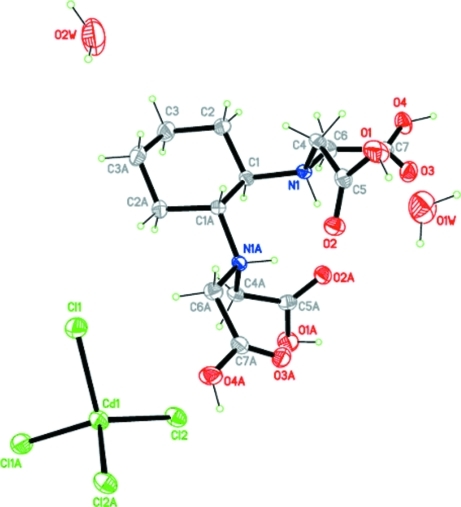
View of the molecular structure of (I), showing the atom-labelling scheme. Displacement ellipsoids are drawn at the 35% probability level. Symmetry related atoms are labeled A (symmetry code: -x, y, -z - 3/2) for the CdCl4 unit and (1 - x,y, -z - 1/2) for the cation.

**Fig. 2.**
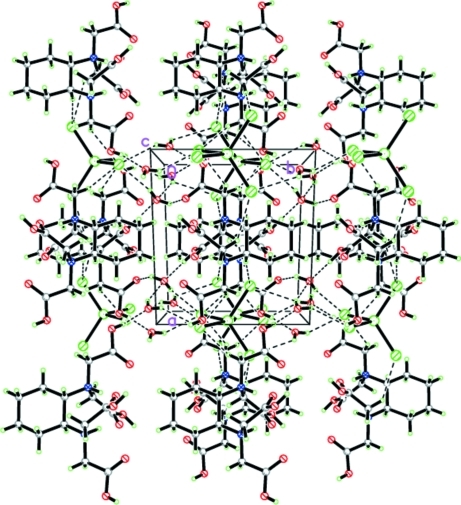
View of the 3-D network for compound (I) along the b axis.

## supplementary crystallographic information

### Comment

In recent years, one-, two- and three-dimensional infinite supramolecular coordination assemblies of Cd(II) have been the subject of great interest owing to their potential applications in many fields, such as catalysis and optical properties (Wang *et al.*, 2006; Zang *et al.*, 2006). *Trans*-1,2- cyclohexanediamine-*N*,*N*,*N*',*N*'-tetra-acetic acid (H_4_CTA) is a multifunctional ligand that not only can coordinate to metal ions to form coordination complexes, also can act as hydrogen bonding donors in forming supramolecular coordination assemblies (Ben Amor & Jouini, 1999; Seibig & Van Eldik, 1998; Wang *et al.*, 1999). In this work, we report a novel Cd(II) complex accidently obtained by CdCl_2_ and H_4_CTA, [CdCl_4_].H_6_CTA.4H_2_O (I).

The molecular structure of the compound (I) is revealed in Fig. 1. The asymmetric unit of the complex consists of 1/2 [CdCl_4_]^2-^ tetrahedral anion unit, one protonated H_6_CTA cation plus two interstitial water molecules. The Cd(II) atom in the anion is tetrahedrally coordinated by four chlorine atoms, in which the bond length of Cd—Cl lie in the range from 2.4465 (6) Å to 2.4725 (7) Å, and the bond angles Cl—Cd—Cl vary from 101.26 (3) to 114.62 (3)°. The cadmium atom in the tetrahedral anion unit, [CdCl_4_]^2-^, lies on a crystallographic rotation axis (site symmetry 2), and the diprotonated organic molecule, [H_6_CTA]^2+^, has a twofold rotation symmetry with the crystallographic twofold axis running through the middle of two C—C bonds of the cyclohexane part.

In the crystal structure of the compound (I), classic inter- and intra- molecular O—H···O, N—H···O, O—H···Cl and C—H···Cl hydrogen bonds are observed (Table 1), which link the ammonium cations, [CdCl_4_]^2-^ anions and uncoordinated water molecules into a 3-D hydrogen-bonded network and stabilize the crystal packing, as shown in Fig. 2.

### Experimental

*Trans*-1,2-cyclohexanediamine-*N*,*N*,*N*',*N*' -tetra-acetic acid (0.012 mol, 0.4156 g) and CdCl_2_ (0.0045 mol, 0.8249 g) were dissolved in dilute HCl (10 ml, 1*M*) and the resultant solution was evaporated slowly at *ca* 323 K. The title compound was obtained as block colourless crystals after several days.

### Refinement

The C-bound H atoms were positioned geometrically, with C—H = 0.98 Å and 0.97 Å and refined as riding with *U*_iso_(H) = 1.2*U*_eq_(C) or 1.5*U*_eq_. Atom H*1B* was positioned geometrically and allowed to ride on N1, with N—H = 0.91Å and *U*_iso_(H)= 1.2*U*_eq_(N). The H atoms bonded to carboxyl O atoms were located in a difference Fourier map and refined with O–H distance restraints of 0.85 (2) Å. Water H atoms were located in a difference map and refined with O—H and H···H distance restraints of 0.85 (1) and 1.39 (2) Å, respectively, and with *U*_iso_(H) = 1.5*U*_eq_(O).

### Crystal data

**Table:**

(C_14_H_24_N_2_O_8_)[CdCl_4_]·4H_2_O	*F*(000) = 684
*M**_r_* = 674.63	*D*_x_ = 1.711 Mg m^−^^3^
Monoclinic, *P*2/*c*	Mo *K*α radiation, λ = 0.71070 Å
Hall symbol: -P 2yc	Cell parameters from 4992 reflections
*a* = 11.3772 (14) Å	θ = 3.0–25.4°
*b* = 8.5734 (10) Å	µ = 1.30 mm^−^^1^
*c* = 16.2189 (16) Å	*T* = 291 K
β = 124.119 (6)°	Block, colorless
*V* = 1309.7 (3) Å^3^	0.68 × 0.54 × 0.28 mm
*Z* = 2	

### Data collection

**Table:**

Siemens SMART CCD area-detector diffractometer	2400 independent reflections
Radiation source: fine-focus sealed tube	2319 reflections with *I* > 2σ(*I*)
graphite	*R*_int_ = 0.021
ω scans	θ_max_ = 25.4°, θ_min_ = 3.0°
Absorption correction: multi-scan (*SADABS*; Sheldrick, 1996)	*h* = −13→13
*T*_min_ = 0.472, *T*_max_ = 0.712	*k* = −9→10
12160 measured reflections	*l* = −19→17

### Refinement

**Table:**

Refinement on *F*^2^	Primary atom site location: structure-invariant direct methods
Least-squares matrix: full	Secondary atom site location: difference Fourier map
*R*[*F*^2^ > 2σ(*F*^2^)] = 0.025	Hydrogen site location: inferred from neighbouring sites
*wR*(*F*^2^) = 0.069	H atoms treated by a mixture of independent and constrained refinement
*S* = 1.01	*w* = 1/[σ^2^(*F*_o_^2^) + (0.0429*P*)^2^ + 0.6848*P*] where *P* = (*F*_o_^2^ + 2*F*_c_^2^)/3
2400 reflections	(Δ/σ)_max_ = 0.002
166 parameters	Δρ_max_ = 0.33 e Å^−^^3^
9 restraints	Δρ_min_ = −0.59 e Å^−^^3^

### Special details

**Table:**

Geometry. All e.s.d.'s (except the e.s.d. in the dihedral angle between two l.s. planes) are estimated using the full covariance matrix. The cell e.s.d.'s are taken into account individually in the estimation of e.s.d.'s in distances, angles and torsion angles; correlations between e.s.d.'s in cell parameters are only used when they are defined by crystal symmetry. An approximate (isotropic) treatment of cell e.s.d.'s is used for estimating e.s.d.'s involving l.s. planes.
Refinement. Refinement of *F*^2^ against ALL reflections. The weighted *R*-factor *wR* and goodness of fit *S* are based on *F*^2^, conventional *R*-factors *R* are based on *F*, with *F* set to zero for negative *F*^2^. The threshold expression of *F*^2^ > σ(*F*^2^) is used only for calculating *R*-factors(gt) *etc*. and is not relevant to the choice of reflections for refinement. *R*-factors based on *F*^2^ are statistically about twice as large as those based on *F*, and *R*- factors based on ALL data will be even larger.

### Fractional atomic coordinates and isotropic or equivalent isotropic displacement parameters (Å^2^)

**Table:**

	*x*	*y*	*z*	*U*_iso_*/*U*_eq_	
Cd1	0.0000	0.53761 (3)	0.2500	0.03615 (11)	
Cl1	0.02889 (8)	0.72055 (7)	0.37802 (5)	0.04979 (18)	
Cl2	0.21701 (5)	0.38349 (7)	0.32009 (4)	0.03949 (15)	
O1	0.4348 (2)	0.2419 (2)	0.94146 (13)	0.0498 (5)	
H1C	0.3671	0.1650	0.9099	0.075*	
O1W	0.2453 (3)	0.0262 (3)	0.8865 (2)	0.0679 (6)	
O2	0.40158 (16)	0.24536 (18)	0.79129 (11)	0.0359 (4)	
O2W	−0.0832 (3)	0.9342 (3)	0.8284 (2)	0.0894 (9)	
O3	0.75562 (17)	0.18174 (18)	0.96741 (12)	0.0415 (4)	
O4	0.96749 (15)	0.3009 (2)	1.05918 (12)	0.0402 (4)	
H4C	0.9916	0.2307	1.1010	0.060*	
N1	0.62609 (17)	0.44661 (18)	0.85909 (12)	0.0222 (3)	
H1B	0.6007	0.3609	0.8194	0.027*	
C1	0.5801 (2)	0.5898 (2)	0.79036 (14)	0.0247 (4)	
H1A	0.6297	0.5854	0.7568	0.030*	
C2	0.6258 (3)	0.7396 (2)	0.85186 (17)	0.0363 (5)	
H2A	0.5848	0.7420	0.8905	0.044*	
H2B	0.7283	0.7402	0.8980	0.044*	
C3	0.5796 (3)	0.8841 (3)	0.78646 (19)	0.0443 (6)	
H3A	0.6088	0.9769	0.8277	0.053*	
H3B	0.6248	0.8856	0.7505	0.053*	
C4	0.5549 (2)	0.4321 (2)	0.91397 (15)	0.0270 (4)	
H4A	0.6261	0.4184	0.9846	0.032*	
H4B	0.5022	0.5267	0.9052	0.032*	
C5	0.4558 (2)	0.2950 (3)	0.87470 (16)	0.0301 (4)	
C6	0.7845 (2)	0.4427 (2)	0.92917 (16)	0.0306 (5)	
H6A	0.8157	0.5296	0.9752	0.037*	
H6B	0.8270	0.4539	0.8917	0.037*	
C7	0.8335 (2)	0.2919 (3)	0.98686 (15)	0.0306 (5)	
H2WA	−0.126 (7)	0.924 (6)	0.7667 (17)	0.18 (3)*	
H2WB	−0.090 (5)	1.020 (3)	0.848 (3)	0.106 (18)*	
H1WA	0.271 (5)	−0.061 (4)	0.915 (4)	0.15 (2)*	
H1WB	0.189 (5)	0.069 (4)	0.899 (6)	0.29 (5)*	

### Atomic displacement parameters (Å^2^)

**Table:**

	*U*^11^	*U*^22^	*U*^33^	*U*^12^	*U*^13^	*U*^23^
Cd1	0.03114 (15)	0.04045 (17)	0.03324 (15)	0.000	0.01584 (12)	0.000
Cl1	0.0697 (4)	0.0412 (3)	0.0407 (3)	0.0038 (3)	0.0323 (3)	−0.0005 (3)
Cl2	0.0280 (3)	0.0544 (4)	0.0334 (3)	0.0020 (2)	0.0156 (2)	−0.0005 (2)
O1	0.0634 (11)	0.0554 (11)	0.0410 (10)	−0.0215 (9)	0.0357 (9)	−0.0021 (8)
O1W	0.0683 (14)	0.0591 (14)	0.0808 (16)	−0.0126 (11)	0.0445 (14)	0.0116 (12)
O2	0.0398 (8)	0.0370 (8)	0.0317 (8)	−0.0104 (7)	0.0205 (7)	−0.0036 (7)
O2W	0.0851 (18)	0.0461 (13)	0.0653 (16)	0.0025 (13)	−0.0017 (14)	0.0128 (12)
O3	0.0376 (9)	0.0303 (8)	0.0397 (9)	−0.0023 (7)	0.0113 (7)	0.0016 (7)
O4	0.0262 (8)	0.0442 (9)	0.0355 (9)	0.0021 (7)	0.0083 (7)	0.0114 (7)
N1	0.0222 (8)	0.0239 (8)	0.0187 (8)	−0.0013 (6)	0.0103 (7)	−0.0016 (6)
C1	0.0271 (10)	0.0240 (9)	0.0206 (9)	−0.0011 (8)	0.0120 (9)	0.0022 (8)
C2	0.0394 (12)	0.0262 (11)	0.0295 (11)	−0.0050 (9)	0.0109 (10)	−0.0033 (9)
C3	0.0488 (14)	0.0257 (11)	0.0440 (13)	−0.0060 (10)	0.0174 (12)	−0.0003 (10)
C4	0.0297 (10)	0.0328 (10)	0.0213 (10)	0.0010 (9)	0.0161 (9)	0.0015 (8)
C5	0.0303 (11)	0.0328 (11)	0.0300 (11)	0.0018 (9)	0.0185 (9)	0.0054 (9)
C6	0.0207 (10)	0.0348 (11)	0.0297 (11)	−0.0005 (8)	0.0101 (9)	0.0033 (9)
C7	0.0288 (11)	0.0341 (11)	0.0241 (10)	0.0006 (9)	0.0119 (9)	−0.0013 (9)

### Geometric parameters (Å, °)

**Table:**

Cd1—Cl2^i^	2.4465 (6)	N1—H1B	0.9105
Cd1—Cl2	2.4465 (6)	C1—C2	1.527 (3)
Cd1—Cl1	2.4725 (7)	C1—C1^ii^	1.536 (4)
Cd1—Cl1^i^	2.4725 (7)	C1—H1A	0.9800
O1—C5	1.314 (3)	C2—C3	1.520 (3)
O1—H1C	0.9209	C2—H2A	0.9700
O1W—H1WA	0.84 (4)	C2—H2B	0.9700
O1W—H1WB	0.86 (7)	C3—C3^ii^	1.508 (5)
O2—C5	1.207 (3)	C3—H3A	0.9700
O2W—H2WA	0.84 (3)	C3—H3B	0.9700
O2W—H2WB	0.82 (3)	C4—C5	1.501 (3)
O3—C7	1.209 (3)	C4—H4A	0.9700
O4—C7	1.304 (3)	C4—H4B	0.9700
O4—H4C	0.8305	C6—C7	1.508 (3)
N1—C6	1.497 (3)	C6—H6A	0.9700
N1—C4	1.508 (2)	C6—H6B	0.9700
N1—C1	1.539 (2)		
			
Cl2^i^—Cd1—Cl2	114.62 (3)	C1—C2—H2B	109.2
Cl2^i^—Cd1—Cl1	110.91 (2)	H2A—C2—H2B	107.9
Cl2—Cd1—Cl1	109.16 (2)	C3^ii^—C3—C2	109.91 (19)
Cl2^i^—Cd1—Cl1^i^	109.16 (2)	C3^ii^—C3—H3A	109.7
Cl2—Cd1—Cl1^i^	110.91 (2)	C2—C3—H3A	109.7
Cl1—Cd1—Cl1^i^	101.26 (3)	C3^ii^—C3—H3B	109.7
C5—O1—H1C	105.8	C2—C3—H3B	109.7
H1WA—O1W—H1WB	110 (3)	H3A—C3—H3B	108.2
H2WA—O2W—H2WB	116 (3)	C5—C4—N1	109.79 (16)
C7—O4—H4C	112.1	C5—C4—H4A	109.7
C6—N1—C4	111.50 (15)	N1—C4—H4A	109.7
C6—N1—C1	110.05 (15)	C5—C4—H4B	109.7
C4—N1—C1	114.62 (15)	N1—C4—H4B	109.7
C6—N1—H1B	106.8	H4A—C4—H4B	108.2
C4—N1—H1B	106.7	O2—C5—O1	125.9 (2)
C1—N1—H1B	106.7	O2—C5—C4	122.82 (18)
C2—C1—C1^ii^	111.29 (14)	O1—C5—C4	111.22 (18)
C2—C1—N1	110.17 (15)	N1—C6—C7	111.04 (17)
C1^ii^—C1—N1	112.08 (13)	N1—C6—H6A	109.4
C2—C1—H1A	107.7	C7—C6—H6A	109.4
C1^ii^—C1—H1A	107.7	N1—C6—H6B	109.4
N1—C1—H1A	107.7	C7—C6—H6B	109.4
C3—C2—C1	111.84 (18)	H6A—C6—H6B	108.0
C3—C2—H2A	109.2	O3—C7—O4	126.6 (2)
C1—C2—H2A	109.2	O3—C7—C6	123.08 (19)
C3—C2—H2B	109.2	O4—C7—C6	110.27 (18)
			
C6—N1—C1—C2	−61.6 (2)	C1—N1—C4—C5	111.04 (18)
C4—N1—C1—C2	65.0 (2)	N1—C4—C5—O2	−26.1 (3)
C6—N1—C1—C1^ii^	173.88 (18)	N1—C4—C5—O1	155.57 (17)
C4—N1—C1—C1^ii^	−59.5 (2)	C4—N1—C6—C7	60.0 (2)
C1^ii^—C1—C2—C3	−53.8 (3)	C1—N1—C6—C7	−171.67 (16)
N1—C1—C2—C3	−178.77 (19)	N1—C6—C7—O3	10.0 (3)
C1—C2—C3—C3^ii^	58.3 (3)	N1—C6—C7—O4	−168.89 (18)
C6—N1—C4—C5	−123.10 (18)		

Symmetry codes: (i) −*x*, *y*, −*z*+1/2; (ii) −*x*+1, *y*, −*z*+3/2.

### Hydrogen-bond geometry (Å, °)

**Table:**

*D*—H···*A*	*D*—H	H···*A*	*D*···*A*	*D*—H···*A*
O1W—H1WA···O3^iii^	0.84 (4)	2.34 (4)	2.970 (3)	132 (5)
N1—H1B···O2	0.91	2.27	2.750 (3)	112
N1—H1B···O2^ii^	0.91	2.04	2.857 (2)	149
O1—H1C···O1W	0.92	1.70	2.590 (4)	162
O2W—H2WA···O1W^iv^	0.84 (3)	2.24 (3)	2.993 (4)	151 (5)
O2W—H2WB···Cl1^v^	0.82 (3)	2.51 (4)	3.144 (3)	136 (4)
O1W—H1WB···Cl1^vi^	0.86 (7)	2.45 (3)	3.227 (3)	152 (6)
O4—H4C···O2W^vii^	0.83	1.75	2.535 (3)	157
C1—H1A···Cl2^viii^	0.98	2.67	3.637 (3)	171
C4—H4A···Cl2^ii^	0.97	2.64	3.600 (2)	170
C4—H4B···Cl2^vi^	0.97	2.83	3.610 (3)	138
C6—H6A···Cl1^ii^	0.97	2.60	3.537 (2)	163

Symmetry codes: (iii) −*x*+1, −*y*, −*z*+2; (ii) −*x*+1, *y*, −*z*+3/2; (iv) −*x*, *y*+1, −*z*+3/2; (v) *x*, −*y*+2, *z*+1/2; (vi) *x*, −*y*+1, *z*+1/2; (vii) −*x*+1, −*y*+1, −*z*+2; (viii) −*x*+1, −*y*+1, −*z*+1.
